# The association of severely worn dentition resulting from betel nut chewing with temporomandibular disorders: a cross-sectional study

**DOI:** 10.1186/s12903-023-03160-2

**Published:** 2023-07-07

**Authors:** Yundong Liu, Tao Yin, Mi He, Changyun Fang, Shifang Peng

**Affiliations:** 1 Health Management Center, Xiangya Hospital, Central South University, 87 Xiangya Road, Changsha, Hunan 410008 China; 2 Changsha Health Vocational College, Changsha, Hunan 410605 China; 3 Department of Stomatology, Xiangya Hospital, Central South University, Changsha, Hunan 410008 China; 4 Department of Infectious Diseases, Xiangya Hospital, Central South University, 87 Xiangya Road, Changsha, Hunan 410008 China

**Keywords:** Betel nut chewing related severely worn dentition, Age, Gender, Intra-articular temporomandibular disorders, Pain-related temporomandibular disorders

## Abstract

**Background:**

Most studies support parafunctions play an important role in temporomandibular disorders (TMD), whereas the association between tooth wear and TMD remains controversial. Betel nut chewing as a parafunction is popular in South and Southeast Asia. We therefore investigated the association of severely worn dentition resulting from betel nut chewing with TMD.

**Methods:**

A cross-sectional analysis of 408 control participants (male: 380, female: 28, 43.62 ± 9.54 years) and 408 participants with betel nut chewing related severely worn dentition (male: 380, female: 28, 43.73 ± 8.93 years) who received dental and TMD checkup according to Diagnostic Criteria for Temporomandibular Disorders (DC/TMD) in Health Management Center, Xiangya Hospital was performed. Betel nut chewing related severely worn dentition meant all the natural teeth had moderate to severe tooth wear [Tooth Wear Index (TWI) ≥ 2)] including ≥ 2 severe wear teeth (TWI ≥ 3) due to betel nut chewing. Multivariable logistic regression analysis was used.

**Results:**

After adjusting for age, gender, betel nut chewing related severely worn dentition, oral submucosal fibrosis, number of missing teeth, number of dental quadrants with missing teeth, visible third molar and orthodontic history, variables of age, gender and betel nut chewing related severely worn dentition were significant for overall TMD. Multivariable analysis showed betel nut chewing related severely worn dentition was significantly associated with intra-articular TMD [odds ratio and 95% confidence intervals: 1.689 (1.271–2.244), *P* = 0.001] in a betel nut chewing dose-dependent manner.

**Conclusion:**

Betel nut chewing related severely worn dentition was associated with intra-articular TMD.

## Introduction

Tooth wear is an indicator of cumulative parafunctions and oral functions, whereas the association between tooth wear and temporomandibular disorders (TMD) remains controversial. Studies of mandible samples from Aboriginal population or prehistoric human population indicate that tooth wear is associated with temporomandibular joint (TMJ) remodeling or degenerative changes [1–4] whereas no significant correlation between the degree of attrition and the severity of degenerative joint disease was found in mandibles from a contemporary sample [5]. Furthermore, the majority of epidemiological investigations found no or limited association between tooth wear and TMD. Pullinger and Seligman found it was difficult to differentiate TMD patients from nonpatients based on the severity of dental attrition and hence doubted the etiologic role of dental attrition in TMD [6–8]. Even though Tsolka found bruxism patients had a higher prevalence of incisor dentine wear and TMD [9] and comments on tooth wear from the dentist or hygienist were more common in TMD population [10], incisal tooth wear was found to have no association with TMD from different populations [11–13]. However, Oginni and Fathima found severe posterior tooth wear but not generalized tooth wear was significantly associated with pain on palpation and TMJ sound [14, 15].

Parafunctions include bruxism, clenching, gum and khat chewing and so on. Most clinical studies support parafunctions play an important role in TMD [9, 16, 17]. Epidemiological investigations using reported bruxism and clenching diagnosis showed the positive association between these parafunctions with TMD [17]. Longitudinal studies demonstrated self-reported jaw parafunction was a predictor of pain-related TMD [16]. However, some studies doubted the roles of parafunction in maintenance of TMD [13, 17–20]; especially investigations about the association between bruxism with TMD using polysomnography-based diagnosis often showed no positive link [17]. Recently cone beam computed tomography (CBCT) and magnetic resonance image (MRI) investigations on qat chewing population suggested the positive relationship between qat chewing with TMD [21–25]. In addition, most reports about gum chewing also supported the positive association between gum chewing and TMD [26–28], even though Osiewicz [20] and Rao [29] found no effects of chewing habits (betel leaf, tobacco, betel nut and gum) on the incidence of TMJ pain and recently physical therapy was shown to reduce pain for TMD patients [30, 31].

Betel quid chewing is popular in South and Southeast Asia. In mainland China, Hunan Province, 16.2% population consume Betel nut [32]. Even though the association between tooth wear and parafunction with TMD remains controversial, Betel nut chewing not only results in severe dentition wear, which often needs full mouth rehabilitation but also neurological changes [33]. However, the association of betel nut chewing related severely worn dentition with adult TMD is unknown. Diagnostic Criteria for Temporomandibular Disorders (DC/TMD) established by the International Research DC/TMD Consortium Network and the Orofacial Pain Special Interest Group is the most commonly used international standards both for clinics and research [34–37]. In this study, we hypothesized that betel nut chewing related severely worn dentition was associated with TMD in 20–60 years adult urban health checkup population using DC/TMD. We aimed to investigate the association of betel nut chewing related severely worn dentition with TMD.

## Materials and methods

### Subjects and data collection

The cross-sectional study was performed in Health Management Center, Xiangya Hospital, Central South University in Changsha city, Hunan province. A pilot study showed the percentage of overall TMD in the control group and the betel nut chewing related severely worn dentition group were 36.7% and 48.2%, respectively. OpenEpi statistical software (Open Source Epidemiologic Statistics for Public Health) was used to calculate the sample size with a 2-sided confidence level of 95%, a power of 90%, and a 1:1 ratio of cases to controls. The minimum sample size for the control group and the betel nut chewing related severely worn dentition group was 408 respectively, so a total of 408 control participants and 408 participants with betel nut chewing related severely worn dentition were included in this study. Written informed consents were obtained for all participants. All the data collection and procedures were approved by the hospital Ethics Committee (ID: 202103734) and performed according to guidelines.

The betel nut chewing related severely worn dentition participants aged 20–60 years received routine medical, dental and TMD checkup consecutively from 2022-03-14 to 2022-09-14. For betel nut chewing related severely worn dentition group, the inclusion criteria included: (1) All natural teeth had moderate to severe tooth wear [Tooth Wear Index (TWI) ≥ 2)] [38] including ≥ 2 teeth with severe tooth wear (TWI ≥ 3); (2) The participants have regular betel nut chewing history. The exclusion criteria included: (1) developmental abnormality of tooth structure; (2) severe chemical/intrinsic or chemical/extrinsic erosion [38]. The control participants aged 20–60 years received routine medical, dental and TMD checkup from 2022-07-15 to 2022-07-25 and from 2023-01-17 to 2023-02-04. The control group was matched with the betel nut chewing related severely worn dentition group with respect to gender and age groups of 20–29 years, 30–39 years, 40–49 years and 50–60 years (Table 1). For the control group, the inclusion criteria included: (1) The participants did not have betel nut chewing history; (2) The natural teeth may have mild to moderate tooth wear (TWI ≤ 2). The exclusion criteria included: (1) developmental abnormality of tooth structure; (2) severe mechanical/intrinsic attrition, severe mechanical/extrinsic abrasion and moderate to severe chemical/intrinsic or chemical/extrinsic erosion according to Wetselaar [38]. None of the participants had any history of oral facial trauma or rheumatoid arthritis. TMD and dental exam was completed by the first author with 10 years of experience in TMD and orofacial pain management. Clinical exam followed DC/TMD Axis I protocol [34, 37]. The diagnosis of intra-articular TMD included: disc displacement with reduction; disc displacement with reduction with intermittent locking; disc displacement without reduction with limited opening; disc displacement without reduction without limited opening; degenerative joint disease and dislocation [34, 37]. The diagnosis of pain-related TMD included: myalgia, myofascial pain with referral, arthralgia and headaches attributed to TMD [34, 37]. Betel nut chewing history was calculated by multiplying mean daily chewing pieces and chewing years. The diagnostic criteria for oral submucosal fibrosis (OSF) included the presence of palpable fibrous bands or palpable stiffness of a large area of oral mucosa and blanching of a large area of oral mucosa [32]. Visible third molar meant presence of clinically visible third molar. The number of unreplaced non-third molar missing teeth was recorded. The number of dental quadrants with non-third molar missing teeth was also recorded.

**Table 1:** Demographic characteristics. Data are showed as frequencies (proportions)

Age group	Control (n = 408)		Betel nut chewing related severely worn dentition (n = 408)	
	Male [n (%)]	Female [n (%)]	Male [n (%)]	Female [n (%)]
20–29 years	21 (5.15)	0 (0)	21 (5.15)	0 (0)
30–39 years	118 (28.92)	4 (0.98)	118 (28.92)	4 (0.98)
40–49 years	123 (30.15)	7 (1.72)	123 (30.15)	7 (1.72)
50–60 years	118 (28.92)	17 (4.17)	118 (28.92)	17 (4.17)

### Statistics

All data were presented as mean ± standard deviations for continuous variables and percentages for categorical variables. The comparison between groups was performed with unpaired student’s t test or non-parametric test for continuous variables and Chi Square test for categorical variables as appropriate. The variables were quantified as: (gender) 1 for male, 2 for female; (visible third molar), 1 for absence, 2 for presence; and (orthodontic history) 1 for no, 2 for yes. The variable of levels of betel nut chewing was quantified as: 1 for no betel nut chewing, 2 for < 100 pieces*years (low level) and 3 for ≥ 100 pieces*years (high level). Multivariable logistic regression analysis of variables including age, gender, betel nut chewing related severely worn dentition or levels of betel nut chewing, OSF, number of missing teeth, number of dental quadrants with missing teeth, visible third molar and orthodontic history was used to study the association of betel nut chewing related severely worn dentition or different levels of betel nut chewing with overall TMD and intra-articular TMD respectively. The software SPSS 21.0 (SPSS Inc., Chicago, IL, United States) was used for statistical analysis. *P* < 0.05 was considered as statistically significant.

## Results

### Baseline data

The control group included 408 participants with a mean age of 43.62 ± 9.54 years. The betel nut chewing related severely worn dentition group included 408 participants with a mean age of 43.73 ± 8.93 years, in which the average betel nut chewing pieces*years was 179.41 pieces*years and the OSF prevalence rate was 21.81%. The betel nut chewing related severely worn dentition group and the control group had similar levels of age, male ratio, missing tooth prevalence, visible third molar prevalence and orthodontic history prevalence (Table 2).

**Table 2:** Basic clinical characteristics. Data are showed as means ± standard deviation (SD) for continuous variables, or frequencies (proportions) for categorical variables. Comparisons were performed using t test or non-parametric test and Chi square test as appropriate. Temporomandibular disorders TMD. Oral submucosal fibrosis OSF

Variables	Control (n = 408)	Betel nut chewing related severely worn dentition (n = 408)	*P* value
Age (years)	43.62 ± 9.54	43.73 ± 8.93	0.871
Male [n (%)]	380 (93.14)	380 (93.14)	1.000
Betel chewing [n (%)]	0	< 100 pieces*years: 177 (43.38) ≥ 100 pieces*years: 231 (56.62)	
OSF	0	89 (21.81)	
Missing tooth [n (%)]	69 (16.91)	75 (18.38)	0.646
Visible third molar [n (%)]	240 (58.82)	232 (56.86)	0.620
Orthodontic history [n (%)]	2 (0.49)	3 (0.74)	1.000
Overall TMD [n (%)]	148 (36.27)	203 (49.75)	*P* < 0.001, Phi value = 0.136
Intra-articular TMD [n (%)]	147 (36.03)	197 (48.28)	*P* = 0.001, Phi value = 0.124
Pain-related TMD [n (%)]	6 (1.47)	12 (2.94)	*P* = 0.233, Phi value = 0.050

The betel nut chewing related severely worn dentition population had a significantly higher prevalence rate of overall TMD (49.75%) compared to 36.27% of overall TMD in the control population before adjusting the confounders (Table 2). For both control and betel nut chewing related severely worn dentition group, intra-articular TMD was the predominant form of TMD and pain-related TMD was significantly lower (Table 2).

### Association of betel nut chewing related severely worn dentition with overall TMD

In the fully adjusted model, variables of age, gender and betel chewing related severely worn dentition were significant for overall TMD. The odds ratio (OR) and 95% confidence intervals (95% CIs) of presence of betel nut chewing related severely worn dentition was 1.775 (1.337–2.358) (*P* < 0.001) for overall TMD after adjustment (Table 3).

**Table 3:** Multivariable logistic regression analysis on the association of betel nut chewing related severely worn dentition with overall TMD. Data are shown as OR and 95% CIs

Variable	*P* value	OR and 95%CIs	R^2^
Age	0.030	0.983 (0.968–0.998)	0.057
Gender	< 0.001	3.321 (1.840–5.995)	
Betel nut chewing related severely worn dentition	< 0.001	1.775 (1.337–2.358)	
OSF	0.870		
Number of missing teeth	0.758		
Number of dental quadrants with missing teeth	0.471		
Visible third molar	0.229		
Orthodontic history	0.758		

In addition, we found the association of betel nut chewing with overall TMD was in a betel nut chewing dose-dependent manner. The OR and 95% CIs of low and high levels of betel nut chewing for overall TMD was 1.359 (1.153–1.602) (*P* < 0.001) after adjustment (Table 4).

**Table 4:** Multivariable logistic regression analysis on the association of low and high levels of betel nut chewing with overall TMD. Data are shown as OR and 95% CIs

Variable	*P* value	OR and 95%CIs	R^2^
Age	0.036	0.984 (0.968–0.999)	0.053
Gender	< 0.001	3.485 (1.932–6.285)	
Levels of betel nut chewing	< 0.001	1.359 (1.153–1.602)	
OSF	0.942		
Number of missing teeth	0.675		
Number of dental quadrants with missing teeth	0.570		
Visible third molar	0.229		
Orthodontic history	0.764		

### Association of betel nut chewing related severely worn dentition with intra-articular TMD

The betel nut chewing related severely worn dentition population had a significantly higher prevalence rate of intra-articular TMD (48.28%) compared to 36.03% in control group before adjusting the confounders (Table 2). In the adjusted model, age, gender and betel nut chewing related severely worn dentition were significant for intra-articular TMD. The OR and 95% CIs of betel chewing related severely worn dentition was 1.689 (1.271–2.244) (*P* = 0.001) for intra-articular TMD after adjustment (Table 5).

**Table 5:** Multivariable logistic regression analysis on the association of betel nut chewing related severely worn dentition with intra-articular TMD. Data are shown as OR and 95% CIs

Variable	*P* value	OR and 95%CIs	R^2^
Age	0.021	0.982 (0.967–0.997)	0.052
Gender	< 0.001	3.168 (1.769–5.675)	
Betel nut chewing related severely worn dentition	0.001	1.689 (1.271–2.244)	
OSF	0.928		
Number of missing teeth	0.861		
Number of dental quadrants with missing teeth	0.337		
Visible third molar	0.232		
Orthodontic history	0.928		

Furthermore, the association of betel nut chewing with intra-articular TMD was in a betel nut chewing dose-dependent manner. The OR and 95% CIs of low and high levels of betel nut chewing for intra-articular TMD was 1.336 (1.133–1.575) (*P* = 0.001) after adjustment (Table 6).

**Table 6:** Multivariable logistic regression analysis on the association of low and high levels of betel nut chewing with intra-articular TMD. Data are shown as OR and 95% CIs

Variable	*P* value	OR and 95%CIs	R^2^
Age	0.025	0.982 (0.967–0.998)	0.050
Gender	< 0.001	3.323 (1.856–5.951)	
Levels of betel nut chewing	0.001	1.336 (1.133–1.575)	
OSF	0.962		
Number of missing teeth	0.785		
Number of dental quadrants with missing teeth	0.411		
Visible third molar	0.231		
Orthodontic history	0.737		

### Association of betel nut chewing related severely worn dentition with pain-related TMD

The betel nut chewing related severely worn dentition population had an insignificantly greater prevalence rate of pain-related TMD (2.94%) compared to 1.47% in control group (Table 2).

## Discussion

In the present study, we found betel nut chewing related severely worn dentition was significantly associated with overall TMD. The hypothesis is accepted.

TMD is caused by multiple factors. The health checkup population were mainly from the urban citizens and the dental and TMD examination was just one part of the regular health examination, so we could exclude the sample including bias. In this study, we found female was the risk factor for TMD after adjustment, which agrees with previous report of woman’s vulnerability to TMD [39]. Age was negatively associated with TMD in the adjusted model, similar with previous literature [39]. Interestingly, Wang found when the number of missing teeth was smaller but the number of dental quadrants with missing posterior teeth was greater, TMD prevalence increased [39]. However in this study we found both the number of missing teeth and the number of dental quadrants with missing teeth were not significantly associated with overall TMD in the multivariable analysis. Wang’s study focused on the association of tooth loss with TMD [39]. In this report, this sample was mainly male population and the prevalence of tooth loss was not high, so greater sample number may be needed to validated the link between tooth loss and TMD. Orthodontic treatment and presence of visible third molar was also not significantly associated with TMD after adjustment.

In the present study, we found betel nut chewing related severely worn dentition was significantly associated with intra-articular TMD and the association was in a betel nut chewing dose-dependent manner. Recent investigation in skeletal assemblage from the prehistoric site found 40.1% condyles with tooth wear displayed lesions typical of TMJ osteoarthritis (OA), suggesting the strong association between tooth wear and TMJ OA [4]. Chung also found 88% of TMD patients have active shiny facets or scratches on the occlusal surface of splints caused by nocturnal bruxism [40]. Patients with parafunctional habits even had a lower success rate of TMJ arthrocentesis [41] whereas splints may produce therapeutic effects by reducing parafunctional activities [42]. Further the majority of investigations demonstrated the positive role of parafunctions of bruxism, clenching, gum and qat chewing in TMD [16, 17, 24, 27]. Recent MRI findings suggested qat chewing habit may be a risk factor for TMJ osteoarthritis and joint effusion [23, 24]. Similar to these reports, the severely worn dentition due to chronic chewing of betel nut may be associated with increased masticatory function, which may increase TMJ loading indirectly reflected by the significantly increased prevalence rate of intra-articular TMD. However, some studies did not find the positive association between tooth wear, especially of anterior teeth with TMD [11–13]. Yadav showed muscle tenderness, pain on mouth opening and deviation of mandible on mouth opening had a significant relation to dental attrition but other signs of temporomandibular disorders such as joint tenderness, referred pain, joint sounds and limitation of mouth opening had no relation to attrition score [43]. This phenomenon could be explained by the more severely worn dentition due to betel nut chewing when compared to the other forms of parafunctions. In this study, betel nut chewing related severely worn dentition often has severely worn posterior teeth, which agrees with past studies that showed severe posterior teeth wear was significantly associated with TMJ sound [14, 15]. In addition, the association of betel nut chewing with intra-articular TMD in a dose-dependent manner may further validate the association of this parafunctional activity with intra-articular TMD.

In this study, we did not find the significant association between betel nut chewing related severely worn dentition and pain-related TMD. Although Carlsson found tooth wear index and tooth grinding at night could predict only TMJ clicking but not TMJ symptoms 20 years later [44]; Hirsch [12], Schierz [13] and Pergamalian [45] showed tooth wear was not significantly correlated with TMJ and muscle pain; and Osiewicz [20], Janal [19] and Rao [29] demonstrated no effects of chewing habits on the incidence of TMJ pain, most investigations of parafunctions including bruxism and gum chewing showed the positive association with TMJ symptoms [16, 17, 27]. Contrary to expectation, Pergamalian showed tooth wear and bruxism activity were not significantly correlated TMJ and muscle pain and even bruxism activity was associated with less pain in the TMJ on palpation [45]. The reasons why betel nut chewing related severely worn dentition was not significantly associated with pain-related TMD should be considered form these aspects. First, heavy betel nut bruxers are mainly 30–60 years males, intra-articular TMD is the main form of TMD and pain-related TMD in these population is relatively low, so we could not further analyze pain-related TMD subgroup. Second, in comparison with the women’s susceptibility to TMD pain, males may have greater ability to withstand and adapt to the harmful increased parafunctional force, which are reflected by the commonly seen masseter hypertrophy in heavy betel nut bruxers. Third in contrast with qat or gum chewing, heavy betel nut chewing could bring about neurologic effects [33] and may dampen the pain response, which needs more investigations. Further, in contrast with partly central controlled bruxism, although betel nut chewing could induce addiction, the patient could stop chewing to relieve the discomfort of masticatory system but the patient could hardly self-control the activity of bruxism.

Several limitations of the present study should be discussed. First, the main sample in the present study was males. Indeed, females have a higher prevalence of TMD and betel nut chewing is a predominantly male habit, but the role of betel nut chewing related severely worn dentition in TMD in these special population deserved investigation, because betel nut chewing related severely worn dentition often needs full mouth rehabilitation. The prevalence rate of pain-related TMD in 30–60 years males is relatively low, so greater sample number is needed to validate the relationship between betel nut chewing related severely worn dentition and pain-related TMD. Second, there are two components of the betel nut chewing and the worn dentition that may affect the TMD in the study group, but tooth wear is tightly related with betel quid chewing and could be an indirect record of this parafunction, and hence it is often difficult to dissect the precise roles of the parafunction such as the betel nut chewing and the severely worn dentition resulting from betel nut chewing in TMD. It is easy and precise to qualify the betel nut chewing activity so we only semi-quantitatively analyzed the dose-dependent effect of betel nut chewing on TMD. In the present study we only focus on betel nut chewing related severely worn dentition, the comparation of TMD prevalence among different worn dentition groups resulting from different parafunctions such as gum chewing, bruxism deserves further investigation. Third, the TMD diagnosis was based on clinical examination of symptoms and signs, TMJ images such as CBCT or MRI were not taken into consideration, so the analysis about the betel nut chewing related severely worn dentition with TMD subgroups could not be performed. Furthermore, psychological factors were reported to be associated with pain sensitivity and TMD and information about social/psychological factors [36, 37] such as DC/TMD Axis II evaluation should have been added in the present large sample analysis. The health checkup was totally open to the general urban population and well accepted by citizens both from the economic and convenience perspectives and the dental and TMD examination was just one part of the regular medical examination. The investigation about the association of betel nut chewing related severely worn dentition with TMD is hard to carry out in the general population, but the investigation in dental clinic may increase the sampling bias. The sample number from the urban health checkup center was relatively large, and hence they could partly represent the general urban population when the above factors are considered.

## Conclusions

Within the limitations of this study, betel nut chewing related severely worn dentition was associated with intra-articular TMD, the predominant TMD type in 30–60 years male population. Quitting the harmful habits of betel quid chewing not only prevents the dentition wear but also may be beneficial for TMJ health. Moreover, when performing full-mouth rehabilitation of severely worn dentition due to betel nut chewing, temporomandibular joint evaluation and multidisciplinary strategies are necessary.

## Declarations

### Data statement

The datasets generated and/or analysed during the current study are not publicly available due to potential privacy concerns but are available from the corresponding author on reasonable request.

## Abbreviations

TMD: Temporomandibular disorders
TMJ: Temporomandibular joint
OA: Osteoarthritis
DC/TMD: Diagnostic Criteria for Temporomandibular Disorders
TWI: Tooth Wear Index
OSF: Oral submucosal fibrosis
OR: Odds ratio
95% CIs: 95% confidence intervals
CBCT: Cone beam computed tomography
MRI: Magnetic resonance image
